# Preservative Effect of Aqueous and Ethanolic Extracts of the Macroalga *Bifurcaria bifurcata* on the Quality of Chilled Hake (*Merluccius merluccius*)

**DOI:** 10.3390/molecules26123774

**Published:** 2021-06-21

**Authors:** José M. Miranda, Bin Zhang, Jorge Barros-Velázquez, Santiago P. Aubourg

**Affiliations:** 1 Department of Analytical Chemistry, Nutrition and Food Science, School of Veterinary Sciences, University of Santiago de Compostela, Avenida Carvallo Calero, s/n, 27002 Lugo, Spain; josemanuel.miranda@usc.es (J.M.M.); jorge.barros@usc.es (J.B.-V.); 2 Key Laboratory of Health Risk Factors for Seafood of Zhejiang Province, College of Food Science and Pharmacy, Zhejiang Ocean University, No 1, Haida South Road, 1, Lincheng Changzhi, Zhoushan 316022, China; zhangbin@zjou.edu.cn; 3 Department of Food Science and Technology, Marine Research Institute (CSIC), c/E. Cabello 6, 36208 Vigo, Spain

**Keywords:** refrigerated hake, *Bifurcaria bifurcata*, aqueous and ethanolic extracts, microbial activity, lipid damage, quality enhancement

## Abstract

This work addressed the preservative behaviour of different icing media containing extracts from the alga *Bifurcaria bifurcata*. A comparative study of the antimicrobial and antioxidant effects of aqueous and ethanolic extracts of this macroalga was carried out. Whole hake (*Merluccius merluccius*) pieces were stored in ice containing either kind of extract and analysed for quality changes throughout a 13-day storage period. A progressive loss of microbial and biochemical quality was detected in all batches as chilling time increased. A significant inhibitory effect (*p* < 0.05) on microbial activity could be observed as a result of including the aqueous (lowering of psychrotrophic and lipolytic counts and pH value) and ethanolic (lowering of psychrotrophic and lipolytic counts) extracts. Additionally, both kinds of extract led to a substantial inhibition (*p* < 0.05) in the lipid hydrolysis rate (formation of free fatty acids), greater in the case of the batch containing ethanolic extract. Concerning lipid oxidation, a similar inhibitory effect (*p* < 0.05) on the formation of secondary compounds (thiobarbituric acid substances) was noticed in fish specimens corresponding to both alga extracts; however, more (*p* < 0.05) peroxide formation was detected in fish corresponding to the ethanolic extract batch. A preservative effect can be concluded for both kinds of extract; this effect agrees with previous studies reporting the presence of hydrophilic and lipophilic bioactive compounds in *B. bifurcata*.

## 1. Introduction

In Asian countries the consumption of seaweeds or macroalgae dates back to ancient times. Interestingly, their consumption has increased in recent years in Western countries due to the search for new sustainable sources of healthy food and natural products [1]. Seaweeds have been shown to be a relevant source of beneficial constituents such as lipids, vitamins, trace minerals, dietary fibre, and amino acids [2,3]. Additionally, seaweeds have attracted great attention because they contain a profitable variety of chemical components with potential antimicrobial [4,5] and antioxidant [6,7] activity.

Icing is the most common method adopted for fish preservation. However, due to the limited shelf life of marine species, it has been applied in combination with other preservation strategies such as slurry ice [8], ozone [9], packaging [10], irradiation [11], high pressure [12], and the addition of natural compounds [13]. Recently, novel icing media incorporating extracts obtained from natural sources, such as organic acids [14,15] or plant extracts [16,17] with a proven preservative effect, have been used with a view to enhancing the shelf life of fish. Based on different studies, it has been observed that the incorporation of such natural preservatives in the icing medium delays fish spoilage and enhances fish quality due to their antimicrobial and antioxidant properties.

Among the recently proposed natural sources, seaweed extracts provide an interesting possibility to enhance seafood quality. According to the European Council Regulation [18], algae are considered food or food ingredients, so their use in food technology should not constitute any hazard to human health. Thus, different kinds of seaweed extract have been shown to enhance fish quality and increase fish shelf life when included in the icing [19], glazing [20], and canning [21] medium, or as a dipping treatment prior to subsequent chilled storage [22]. Previous research has shown that the extract yield and the preservative compound content obtained from algae can be influenced by the extraction procedure [23,24]. Thus, alcoholic solvents are better for obtaining a higher level of phenolic compounds [6,25], while water extraction is especially convenient for obtaining high yields of active polysaccharides, proteins, and peptides [26,27]. Studies concerning the comparative effects of aqueous and alcoholic seaweed extracts on seafood quality are scarce. Thus, Barros-Velázquez et al. [28] studied the storage of hake (*Merluccius merluccius*) in an icing medium containing aqueous and ethanolic extracts of *Fucus spiralis*. They observed a remarkable antimicrobial and antioxidant effect in fish corresponding to the ethanolic extract ice, while aqueous extracts led to a negligible preservative effect. It was concluded that further comparative studies of the effects of both kinds of extracts on seafood would be necessary.

Among brown seaweeds, *Bifurcaria bifurcata* represents an interesting choice on the basis of its great availability in the Atlantic coast of France, Spain, Portugal, Ireland, and the United Kingdom [29,30]. Interestingly, previous studies have shown the presence of hydrophilic (phlorotannins, phenolic acids, flavonoids, alginates, polisaccharides in general) [31,32,33] and lipophilic (polyphenols and sterols) [34,35,36] preservative compounds in *B. bifurcata*. This work addresses the comparative preserving effects of aqueous and ethanolic extracts of this macroalga. For it, icing media containing either kind of alga extract were prepared and applied to whole hake *(M. merluccius*) specimens that were stored for 13 days. The evolution of microbial and chemical quality indices was comparatively analyzed throughout chilled storage.

## 2. Results and Discussion

### 2.1. Evolution of Microbial Development during Chilled Storage of Hake

**Figure 1:**
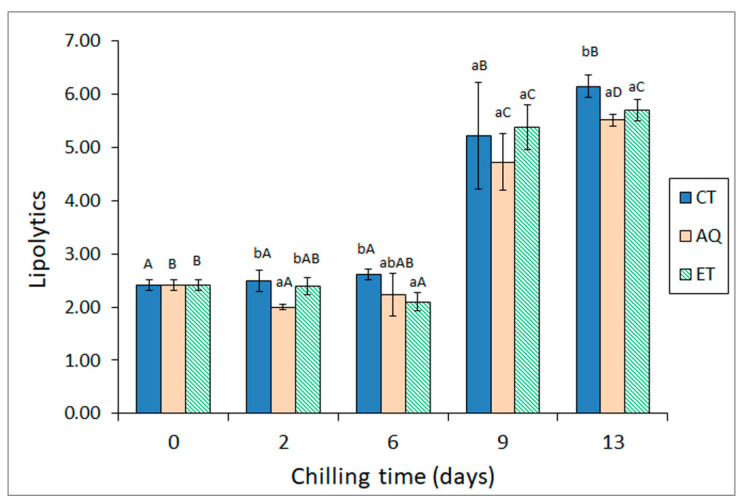
Evolution of lipolytic counts (log CFU·g^−1^) * in chilled hake subjected to different icing conditions **. * Average values of three replicates (*n* = 3); standard deviations are indicated by bars. For each chilling time, different lowercase letters denote significant (*p* < 0.05) differences as a result of icing conditions. For each icing condition, different capital letters denote significant (*p* < 0.05) differences as a result of chilling time. ** Icing conditions as expressed in Table 1.

The results of the microbiological analysis for all three batches during refrigerated storage are presented in Table 1 and Figure 1. With respect to the aerobe count, a widely used index of microbial quality, the results indicated a slight protective effect of the aqueous alga extract on fish quality. Thus, and although differences among batches were not significant (*p* > 0.05), the batch containing the aqueous extract exhibited better control (i.e., lower average counts) of aerobe growth at medium and advanced storage times (Table 1). Remarkably, none of the three batches reached 7 log CFU·g^−1^, generally considered as a limit of acceptability for seafood products [37].

With respect to psychrotrophs, the control batch surpassed 6 log CFU·g^−1^ on day 9, while the counterpart batches containing alga extracts were below that value at that time (Table 1). Remarkably, the inclusion of alga extract, either aqueous (AQ batch) or ethanolic (ET batch), provided a slight protection of fish muscle with respect to psychrotrophs’ growth for the 6–13-day period (i.e., lower average counts), although this effect was found to be statistically significant (*p* < 0.05) only at advanced storage times (day 13).

Table 1 also provides the comparative analysis of *Enterobacteriaceae* growth in all three batches. The presence of this bacterial group was very limited in all three batches, with microbial counts being always below 2 log CFU·g^−1^ (Table 1).

Proteolytic bacteria can cause the breakdown of fish muscle structure by biosynthesising and secreting extracellular proteases, whose effect on the myofibrillar protein fraction negatively affects fish texture and quality. As in the case of aerobes, the presence of alga extract in the icing medium provided, in general terms, a slight protective effect on fish muscle (Table 1). The average counts of proteolytic bacteria in the AQ batch were slightly below those determined in the control batch, especially at medium and advanced storage times (day 6 and onwards), although such differences were not significant (*p* > 0.05). As in the case of aerobes and psychrotrophs, the AQ extract seemed to provide better protection as compared to the ET extract.

The comparative evolution of lipolytic bacteria in all three batches is displayed in Figure 1. This microbial group is able to biosynthesise and secrete extracellular lipases whose effect on triacylglycerides (TG) and phospholipids (PL) negatively affects fish quality. In our study, the inclusion of alga extract in the icing medium resulted in more limited growth of these specific spoilage organisms at different storage times. Thus, the AQ extract provided a significant (*p* < 0.05) reduction in lipolytic bacteria at both early (day 2) and advanced (day 13) storage times. With respect to the ET extract, this beneficial effect was significant (*p* < 0.05) for medium (day 6) and advanced (day 13) storage. Remarkably, neither AQ nor ET batches reached 6 log CFU·g^−1^ even on day 13, while the control batch surpassed this value at that time. As was also observed for aerobes, psychrotrophs, and proteolytic bacteria, the AQ extract provided, in general terms, better control of the growth of lipolytic bacteria as compared to the ET extract.

This study has proven the antimicrobial effect on hake muscle of including aqueous and ethanolic *B. bifurcata* extracts in the icing media. This inhibition of microbial growth could be explained by the presence of different kinds of bioactive compounds and their antimicrobial activity due to several mechanisms, such as the inhibition of extracellular microbial enzymes, deprivation of substrates required for microbial growth, direct action on the microbial metabolism through inhibition of oxidative phosphorylation, and complexation of metal ions in the bacterial growth environment [4,38]. Concerning the bioactive antimicrobial components present in *B. bifurcata*, several previous studies have reported their presence both in aqueous and lipophilic alga extracts.

Related to *B. bifurcata* lipophilic extracts (Table 2), previous research showed the presence of bioactive compounds such as phenols in aq. 80% ethanol extract [39] and diterpenes in ethyl ether extract [40]. Furthermore, total sterol content was analysed by FTIR spectroscopy in methanol:chloroform extracts (1:1) by Bouzidi et al. [35], fucosterol being identified as the major sterol. Additionally, a substantial antimicrobial effect was reported in methanolic extracts of this macroalga by Alves et al. [36] on the basis of its high content in polyphenolic compounds, evaluated by in vitro tests (DPPH and ORAC assays). Moreover, ethanolic extracts of the algae *B. bifurcata* [19] reduced the microbial counts (aerobes, psychrotrophs, proteolytic and lipolytic bacteria, and *Enterobacteriaceae*) in chilled megrim (*Lepidorhombus whiffiagonis*) muscle during storage.

Previous research on *B. bifurcata* aqueous extracts has also reported the presence of different kinds of antimicrobial components (Table 2). Thus, Gómez-Ordóñez and Rupérez [32] identified alginate as the main polysaccharide by FTIR-ATR. Later on, dietary fibre and physicochemical properties of *B. bifurcata* were studied by Gómez-Ordóñez et al. [31]. As a result, total dietary fibre content of this alga was 37.42% of which 14.64% was soluble, while insoluble fibres represented 22.79%; notably, the soluble fibre contained uronic acids from alginates and neutral sugars from sulphated fucoidan and laminarin, insoluble fibres being essentially made from cellulose. Recently, Agregán et al. [33] analysed the phenolic compounds in this kind of extract and identified phlorotannins as the main phenolic compounds, these being followed by phenolic acids, flavonoids, fuhalols, hydroxyfuhalols, eckol derivatives, and rosmarinic acid.

Concerning the preservative effects on seafood developed by other macroalgae, ethanolic extracts of the alga *U. pinnatifida* [41] reduced the microbial activity (aerobes, psychrotrophs, proteolytic and lipolytic bacteria, and *Enterobacteriaceae* counts) in chilled megrim (*Lepidorhombus whiffiagonis*) muscle during storage. Remarkably, stronger inhibition of the development of microbes (aerobes, psychrotrophs, *Enterobacteriaceae*, and proteolytic and lipolytic bacteria) was observed in chilled hake stored in an icing system containing an ethanolic extract of *Fucus spiralis* as compared with a counterpart fish batch stored in ice containing an aqueous extract of this alga [28].

Previous studies have also reported on the enhancement of microbial quality in chilled fish by including aqueous or ethanolic extracts obtained from plants in the icing medium. Thus, the inclusion of ethanolic extracts of thyme (*Thymus vulgaris*), oregano (*Origanum glandulosum*), or clove (*Syzygium aromaticum*) led to lower average values for mesophilic aerobic and psychrotrophic bacteria in chilled anchovy (*Engraulis entresols*) muscle [42]. Likewise, a reduction in total viable bacteria and an increase in shelf life resulted from the inclusion of ethanolic mint (*Mentha arvensis*) leaf or citrus (*Citrus aurantium*) peel extracts in ice during the storage of Indian mackerel (*Rastrelliger kanagurta*) [43]. Concerning aqueous extracts, the presence in the fish storage ice of a rosemary (*Rosmarinus officinalis*) extract [17] provoked a reduction in total viable bacteria and an increased shelf life in chilled sardine (*Sardinella aurita*). Additionally, inhibition of total viable counts and increased sensory acceptance were observed in chilled mackerel (*Rastrelliger kanagurta*) as a result of the presence of an aqueous extract from two garcinia species (*Garcinia indica* and *G. cambogia*) [44].

### 2.2. Comparative Evolution of pH Value and Free Fatty Acid (FFA) Content during Chilled Storage of Hake

**Figure 2:**
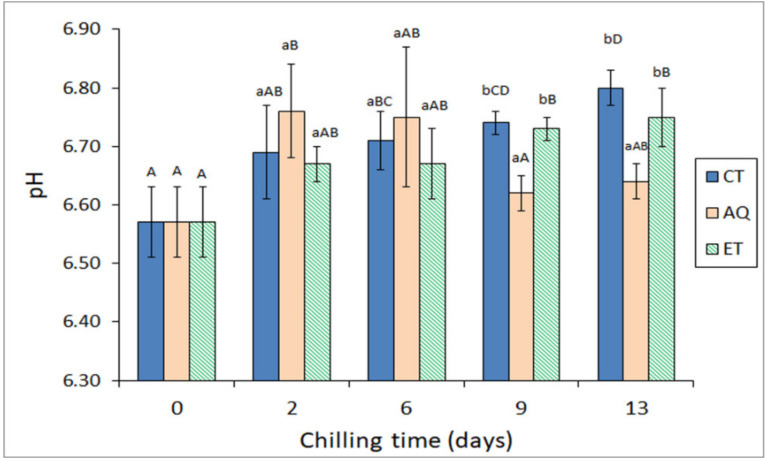
Evolution of pH value * in chilled hake subjected to different icing conditions **. * Average values of three replicates (*n* = 3); standard deviations are indicated by bars. For each chilling time, different lowercase letters denote significant (*p* < 0.05) differences as a result of icing conditions. For each icing condition, different capital letters denote significant (*p* < 0.05) differences as a result of chilling time. ** Icing conditions as expressed in Table 1.

As shown in Figure 2, the pH value of fish muscle increased (*p* < 0.05) with storage time in specimens corresponding to control (CT) and ET batches. On the contrary, hake stored under AQ icing conditions exhibited negligible differences as storage time progressed. Thus, fish specimens corresponding to the AQ batch showed lower (*p* < 0.05) pH values than their counterparts from CT and ET batches in the 9–13-day period. Consequently, an inhibitory effect of the aqueous alga extract on the pH increase could be inferred. Increases in the pH value of fish muscle during storage have been reported to indicate the accumulation of alkaline compounds, such as ammonia, trimethylamine, and other nitrogen-containing compounds, which are mainly derived from microbial spoilage [4]. The present inhibition of a pH increase can be explained on the basis of the above-mentioned antimicrobial compounds (Section 2.1; Table 2) present in both kinds of *B. bifurcata* extracts.

A different result was reported by Barros-Velázquez et al. [28] when comparing the effects of two ice systems containing aqueous and ethanolic extracts of the alga *F. spiralis*, respectively. In their study, no differences in pH value were found between batches of chilled megrim (*L. whiffiagonis*) muscle; in addition, no differences were observed as compared with fish specimens of the control batch which did not contain any alga extract. In contrast, the results obtained in another study allowed the conclusion that including ethanolic *B. bifurcata* extract in the icing system for megrim (*L. whiffiagonis*) muscle led to better maintenance of the pH value as compared with the control batch during chilled storage [19].

In agreement with the current results, previous studies have reported an inhibition of the pH increase in chilled fish as a result of using other natural preservative compounds in the icing medium. These studies used aqueous extracts obtained from rosemary (*R. officinalis*) and applied to chilled sardine (*S. aurita*) [17], and from garcinia (*G. indica* and *G. cambogia*) employed for mackerel (*R. kanagurta*) refrigeration [44]. Notably, previous research on plant extracts has also shown an inhibition of an increased pH value of chilled fish specimens when using ethanol extracts from thyme (*T. vulgaris*), oregano (*O. glandulosum*), or clove (*S. aromaticum*) on anchovy (*E. encrasicolus*) [42] and from mint (*M. arvensis*) leaf and citrus (*C. aurantium*) leaf extracts during the chilled storage of Indian mackerel (*R. kanagurta*) [43].

A substantial increase (*p* < 0.05) in FFA content was detected in all batches as storage time progressed (Table 3). A comparison among batches showed lower average values in specimens corresponding to the ET batch; differences were found to be significant (*p* < 0.05) throughout the whole experiment as compared with the control batch and for the 2–9-day period as compared to the AQ batch. Notably, FFA formation was also inhibited in fish corresponding to the AQ batch at days 2, 6, and 13.

Both endogenous and microbial lipases have been reported to be responsible for the formation of FFA during the chilled storage of fish [8]. Before the end of the microbial lag phase (about 6–9 days), endogenous enzyme activity should be predominant; after that time, microbial activity should gain importance and be mostly responsible for the development of lipid hydrolysis. The results of the present study show a constant and gradual increase in FFA content with chilling time, so it can be concluded that both mechanisms occurred. Moreover, the results obtained can be considered the result of two opposite reactions. On one side, both endogenous and microbial enzymes (namely, lipases and phospholipases) can hydrolyse high-molecular-weight lipids such as PL and TG. On the other, and as they are low-molecular-weight molecules, FFA are likely to be rapidly oxidised or broken down during storage due to their greater accessibility to oxygen and other pro-oxidant molecules as compared to TG and PL [45]. On the basis of the marked formation of FFA, the first factor has been shown to be more important.

The current inhibitory effect on FFA formation in hake muscle stored under both ice batches can be explained on the basis of the presence of antimicrobial compounds present in both kinds of *B. bifurcata* extracts. Consequently, the above-mentioned bioactive compounds (Section 2.1; Table 2) can be involved in such a preserving effect. In agreement with the current results, inhibition of FFA formation derived from the inclusion of an ethanolic extract of *B. bifurcata* in the icing medium was observed in chilled megrim (*L. whiffiagonis*) by Miranda et al. [19]. A similar inhibitory effect on FFA formation was observed when an ethanolic extract of *U. pinnatifida* was employed for the chilled storage of megrim (*L. whiffiagonis*) [37]. Furthermore, FFA formation showed to be diminished during the storage of hake (*M. merluccius*) when an icing system containing the alga *F. spiralis* was employed [28]; remarkably and according to the current study, a lower FFA formation was observed in chilled hake stored in ice containing an ethanolic alga extract as compared to its counterpart kept in an icing system containing an aqueous extract. 

In agreement with the inhibition of FFA formation reported in the present study, previous research has shown the effect of including plant extracts in the icing medium of fish. Thus, a lower FFA content was detected in anchovy (*E. encrasicolus*) due to the presence of an ethanolic thyme (*T. vulgaris*), oregano (*O. glandulosum*), or clove (*S. aromaticum*) extract [40] in the icing medium. A similar situation concerning FFA formation was observed in Indian mackerel (*R. kanagurta*) due to the presence of ethanolic mint (*M. arvensis*) leaf and citrus (*C. aurantium*) leaf extracts [43] in the icing medium. In agreement with the results of the present study, Quitral et al. [16] also reported the inhibition of FFA formation due to the inclusion of aqueous oregano (*O. vulgare*) or rosemary (*R. officinalis*) extract in the ice employed for storage of Chilean jack mackerel (*Trachurus murphyi*).

### 2.3. Lipid Oxidation Development during Chilled Storage of Hake

Progressive formation of peroxides (*p* < 0.05) was observed in all batches as storage time progressed (Table 3). Nevertheless, the values detected were in all cases below 7.5, which can be considered a relatively low level for refrigerated fish [8]. A comparison among batches showed higher (*p* < 0.05) values in fish specimens corresponding to the ethanolic extract batch at all storage times. Notably, fish specimens belonging to the AQ batch exhibited lower average values than the counterpart CT batch (6–13-day period), although differences were not significant (*p* > 0.05).

A significant trend (*p* < 0.05) of an increase in the content of secondary lipid oxidation compounds (i.e., thiobarbituric acid reactive substances, TBARS) was observed for fish specimens corresponding to CT and AQ batches (Table 3). Concerning the ET batch, average values showed an increase up to day 6, this being followed by a decrease until the end of the storage time. A comparison among batches showed an inhibitory effect (*p* < 0.05) of the aqueous alga extract (AQ batch) at days 2 and 13, while this effect was only detected at day 13 in fish belonging to the ET batch.

It is concluded that the inclusion of a water extract of *B. bifurcata* in the icing medium employed for the chilled storage of hake led to the inhibition of lipid oxidation events (i.e., the formation of secondary compounds) in hake muscle. This effect can be explained by the presence of relatively polar compounds (namely, hydrophilic molecules), which have been reported to play a crucial role in the inhibition of oxidation [46,47]. With respect to the ethanol extract, no definite effect could be inferred, as increased peroxide formation was accompanied by a decrease in TBARS.

Previous research has accounted for the presence of antioxidant compounds in the aqueous extract of *B. bifurcata* (Table 2). Thus, the dietary fibre and physicochemical properties of *B. bifurcata* were studied by Gómez-Ordóñez et al. [31]. As a result, total dietary fibre content was 37.42% of which 14.64% was soluble, insoluble fibres representing 22.79% of the content; notably, the soluble fibre contained uronic acids from alginates and neutral sugars from sulphated fucoidan and laminarin, while insoluble fibres were essentially made from cellulose. Furthermore, Gómez-Ordóñez and Rupérez [32] identified alginate by FTIR-ATR as the main polysaccharide in *B. bifurcata* aqueous extract. Agregán et al. [33] concluded that *B. bifurcata* aqueous extracts could be used as sources of antioxidant phenolic compounds on the basis of the presence of phlorotannins, phenolic acids, flavonoids, fuhalols, hydroxyfuhalols. The same research group [48] showed antioxidant activity (DPPH, FRAP, and ORAC assays) and also an antioxidant effect on canola oil during storage at 60 °C by the presence of the aqueous *B. bifucata* extract. 

In agreement with the results of the present study, less peroxide formation was also observed in hake (*M. merluccius*) muscle subjected to an ice system containing an aqueous extract of *F. spiralis* as compared with its counterpart treated with an ethanolic extract [28]. Furthermore, ethanolic extracts of *B. bifurcata* [19] led to higher levels of peroxides in chilled megrim (*L. whiffiagonis*), while the TBARS content was not modified as compared to the control fish batch. Neither did an ethanolic extract of *U. pinnatifida* provide significant differences in lipid oxidation rates when included in an icing medium for the chilled storage of megrim (*L. whiffiagonis*) [41]. However, greater rancidity stability was detected in a fish oil system in the presence of different kinds of ethanolic macroalga extracts when compared to their counterpart aqueous extracts [25]. Concerning thermally treated fish, an inhibitory effect of aqueous alga extracts was also detected by Ortiz et al. [21] in canned fish. Thus, aqueous extracts of various algae (*Durvillaea antarctica*, *Ulva lactuca*, *Pyropia columbina*, *Macrocystis pyrifera*, and *Gracilaria chilensis*) were included in the packaging medium during the canning of Atlantic salmon (*Salmo salar*); as a result, remarkable rancidity stabilisation (i.e., *p*-anisidine value) was observed throughout an accelerated canned storage study (up to 140 days at 40 °C).

Previous research has also shown the inhibition of lipid oxidation in fish muscle derived from the presence of aqueous or ethanolic plant extracts. Thus, less formation of peroxides and TBARS was detected in mackerel (*R. kanagurta*) in the presence of aqueous garcinia (*G. indica* or *G. cambogia*) extracts in the icing medium [44], as well as in Chilean jack mackerel (*Trachurus murphyi*) due to the presence of aqueous oregano (*O. vulgare*) or rosemary (*R. officinalis*) extract in the icing medium [16]. Moreover, a substantial reduction in both primary and secondary lipid oxidation events was observed when ethanolic extracts of thyme (*T. vulgaris*), oregano (*O. glandulosum*), or clove (*S. aromaticum*) were used for the chilled storage of anchovy (*E. encrasicolus*) [42]. A similar result was observed in chilled Indian mackerel (*R. kanagurta*) when stored in the presence of ethanolic extracts of mint (*M. arvensis*) leaf and citrus (*C. aurantium*) leaf [43].

## 3. Materials and Methods

### 3.1. Starting Macroalga B. bifurcata and Preparation of Icing Systems

The lyophilised alga *B. bifurcata* was provided by Porto-Muiños (Cerceda, A Coruña, Spain). Two kinds of alga extract were prepared.

As a first preparation, 15 g of lyophilised alga were distributed into 3 tubes (5 g in each) and mixed with distilled water (40 mL in each), stirred for 30 s, centrifuged at 2500× *g* for 10 min at 4 °C, and the supernatants recovered. Then, the remaining alga sediments were extracted a second time with the same water quantities. Finally, all supernatants were recovered and diluted to 6 L with 250 mL of absolute ethanol and distilled water (2.50 g lyophilised alga L^−1^ aqueous solution). This solution was packaged in polyethylene bags, kept frozen at −18 °C, and later used as an icing medium (AQ batch).

Similarly, 15 g of lyophilised alga were distributed into 3 tubes (5 g in each) and mixed with absolute ethanol (40 mL in each), stirred for 30 s, centrifuged at 2500× *g* for 10 min at 4 °C, and the supernatants recovered. Then, the remaining alga sediments were extracted a second time with the same absolute ethanol quantities and supernatants recovered. All supernatants were made up to 250 mL with absolute ethanol and finally diluted to 6 L with distilled water (2.50 g lyophilised alga L^−1^ aqueous solution). This solution was also packaged in polyethylene bags, kept frozen at −18 °C, and later used as icing medium (ET batch).

Finally, 250 mL of absolute ethanol were diluted in 6 L of distilled water. The solution was packaged, kept frozen in the same way as the two other ices, and referred to as the control (CT batch).

Before the addition to individual fish specimens, the different icing systems were ground to obtain ice flakes. Experimental conditions (namely, the content of lyophilised alga extract in the ice) employed in the present study were based on previous research carried out at our laboratory, as described elsewhere [19]. pH values in the solutions employed for preparing the three icing media were 5.79 ± 0.05, not being influenced by the presence of the alga extract.

### 3.2. Evolution of Microbial Development during Chilled Storage of Hake

Fresh hake (78 specimens) were caught near the Galician Atlantic coast (North–Western Spain) and transported to the laboratory. Throughout this process (10 h), the fish specimens were maintained in ice. The length and weight of the fish specimens ranged from 30.0 to 31.5 cm and from 185 to 215 g, respectively.

Upon arrival at the laboratory, six individual fish specimens were separated and analysed as initial fish (day 0). These fish specimens were divided into three different groups (two individuals per group) that were analysed independently to achieve the statistical analysis (*n* = 3). The remaining fish specimens were divided into three batches (24 individuals in each batch) that were placed in separate boxes and directly surrounded by different kinds of ice (CT, AQ, and ET batches, respectively), prepared as previously described. Ice was added at a 1:1 fish:ice ratio, and all batches were placed inside a refrigerated room (2 ± 1 °C). Boxes that allowed drainage of melted ice were used for fish storage. The ice of all batches was renewed when required to maintain the mentioned fish:ice ratio. All batches were stored for a 13-day period, being sampled and analysed on days 2, 6, 9, and 13. In order to carry out the statistical analysis, each batch was carried out in triplicate (*n* = 3). At each sampling time, six specimens were taken from each batch (two specimens per replicate) for analysis. Specimens from the same replicate were pooled together and analysed independently from specimens corresponding to other replicates.

### 3.3. Determination of Microbial Development

Hake muscle samples (10 g) were taken aseptically from chilled fillets and homogenised with 90 mL of 0.1% peptone water (Merck, Darmstadt, Germany) in sterile stomacher bags (AES, Combourg, France) as previously described [14,15]. Aerobes were investigated on plate count agar (PCA, Oxoid Ltd., London, UK), incubation being carried out for 48 h at 30 °C. Psychrotrophic bacteria were counted in PCA, after an incubation period of 7 days at 7–8 °C. *Enterobacteriaceae* were investigated in Violet Red Bile Agar (VRBA) (Merck, Darmstadt, Germany) after incubation at 37 ± 0.5 °C for 24 h. Microorganisms able to produce proteolytic or lipolytic extracellular enzymes were determined in casein agar or tributyrin agar, respectively, incubation being carried out for 48 h at 30 °C, as previously reported [49]. The limits of detection of the microbial methods were 10 CFU·g^−1^ in the case of aerobes, psychrotrophs, and *Enterobacteriaceae*, and 100 CFU·g^−1^ for both lipolytic and proteolytic bacteria.

For all microbiological analyses, bacterial numbers were converted into log CFU·g^−1^ muscle before performing the statistical analysis. All analyses were conducted in triplicate.

### 3.4. Assessment of Chemical Indices Related to Quality Loss

The evolution of the pH value in hake muscle during storage was determined in the dorsal fillets of hake specimens using a 6 mm diameter insertion electrode (Crison, Barcelona, Spain).

Lipids from hake white muscle were extracted following the method of Bligh and Dyer [50] in which single-phase solubilisation of the lipids was employed by means of a chloroform–methanol (1:1) mixture. Results were calculated as g lipid·kg^−1^ hake muscle.

The FFA content was determined using the lipid extract of hake muscle according to the method developed by Lowry and Tinsley [51]. This method is based on the formation of a complex between FFA and cupric acetate–pyridine, followed by spectrophotometric determination at 715 nm (Beckman Coulter DU 640 spectrophotometer, Brea, CA, USA). Results were calculated as mg FFA·kg^−1^ lipids.

The peroxide value was determined spectrophotometrically (520 nm) in the lipid extract of hake according to the method developed by Chapman and McKay [52] in which peroxides in the lipid extract are reduced with ferric thiocyanate. Results were calculated as meq. active oxygen·kg^−1^ lipids.

The thiobarbituric acid index (TBA-i) was determined according to the method proposed by Vyncke [53]. This method is based on the reaction between a trichloroacetic acid extract of hake white muscle and thiobarbituric acid. In it, the content of TBARS is spectrophotometrically measured at 532 nm. For quantitative purposes, a standard curve using 1,1,3,3-tetraethoxypropane was employed. Results were calculated as mg malondialdehyde·kg^−1^ muscle.

### 3.5. Statistical Analysis

Data corresponding to microbial and chemical parameters related to quality loss were subjected to ANOVA to explore differences resulting from the effect of icing conditions and chilling time. In all cases, analyses were carried out using PASW Statistics 18 software for Windows (SPSS Inc., Chicago, IL, USA). Comparisons of means were performed using the least-squares difference (LSD) method. Differences among batches were considered significant for a confidence interval at the 95% level (*p* < 0.05).

## 4. Conclusions

A progressive loss of microbial and biochemical quality was detected in all batches as storage time progressed. A similar inhibitory effect (*p* < 0.05) on microbial activity could be observed as a result of including the aqueous (lowering of psychrotrophic and lipolytic counts and pH level) or ethanolic (lowering of psychrotrophic and lipolytic counts) *B. bifurcata* extracts. Additionally, both kinds of extract led to significant inhibition (*p* < 0.05) of lipid hydrolysis events (FFA formation) that was more intense in the case of the ethanol extract batch. Concerning lipid oxidation events, a similar inhibitory effect (*p* < 0.05) on the level of secondary compounds (TBARS formation) was observed in fish specimens corresponding to both alga extracts; however, more (*p* < 0.05) peroxide formation was detected in fish corresponding to the ethanolic extract batch as compared to the aqueous extract.

From these results, a promising preservative effect of both kinds of alga extracts can be concluded. This effect agrees with previous studies showing the presence of hydrophilic and lipophilic bioactive compounds in *B. bifurcata* and, consequently, the need for testing and applying different kinds of extracts. The current results reinforce the role of macroalgae as a source of bioactive compounds in order to preserve seafood quality. This green preservation strategy matches with current global interests in the search for effective antimicrobials and antioxidants from natural sources to replace synthetic preservatives in food.

## Figures and Tables

**Table 1:** Evolution of microbial development (log CFU·g^−1^) * in chilled hake subjected to different icing conditions **.

Microbial Group	Chilling Time (Days)	Icing Condition		
		CT	AQ	ET
**Aerobes**	0	2.52 A (0.24)	2.52 A (0.24)	2.52 A (0.24)
	2	2.35 aA (0.37)	2.77 aAB (0.81)	2.20 aA (0.17)
	6	3.92 aB (0.15)	3.71 aB (0.74)	4.04 aB (0.52)
	9	5.28 abC (0.51)	5.09 aC( (0.46)	5.95 bC (0.32)
	13	5.71 aC (0.59)	5.44 aC (0.25)	5.61 aC (0.48)
**Psychrotrophs**	0	3.38 A (0.41)	3.38 A (0.41)	3.38 B (0.41)
	2	3.20 bA (0.13)	3.56 bA (0.26)	2.20 aA (0.35)
	6	5.44 aB (0.24)	5.34 aB (0.03)	5.40 aC (0.28)
	9	6.30 aC (0.45)	5.91 aC (0.34)	5.97 aC (0.47)
	13	7.81 bD (0.35)	6.80 aD (0.23)	6.97 aD (0.46)
*Enterobacteria-ceae* ***	0	1 (0.0)	1 (0.0)	1 (0.0)
	2	1 (0.0)	1 (0.0)	1 (0.0)
	6	1 (0.0)	1 (0.0)	1 (0.0)
	9	1 (0.0)	1.10 (0.17)	1.66 (0.58)
	13	1.36 (0.32)	1 (0.0)	1 (0.0)
**Proteolytics**	0	2.40 A 0.46)	2.40 A (0.46)	2.40 A (0.46)
	2	2.20 aA 0.17)	2.42 aA (0.39)	2.00 aA (0.33)
	6	3.44 aB (0.10)	3.32 aB (0.15)	3.55 aB (0.33)
	9	5.29 aC (0.08)	5.27 abC (0.49)	5.64 bC (0.03)
	13	6.78 aD (0.47)	6.55 aD (0.23)	6.26 aD (0.43)

* Average values of three replicates (*n* = 3); standard deviations are indicated in brackets. For each chilling time, different lowercase letters denote significant (*p* < 0.05) differences as a result of icing conditions. For each icing condition, different capital letters denote significant (*p* < 0.05) differences as a result of chilling time. ** Icing conditions: CT (control; ice prepared without alga extract), AQ (ice containing an aqueous alga extract), and ET (ice containing an ethanolic alga extract). *** No effect (*p* > 0.05) of icing time or icing condition on *Enterobacteriaceae* counts was detected.

**Table 2:** Previous research related to analysis of bioactive compounds included in hydrophilic and lipophilic extracts of alga *Bifurcaria bifurcata*.

Extraction Medium	Bioactive Compound	Reference
aq. 80% ethanol	phenols	Glombitza et al. 1976 [39]
ethyl ether	diterpenes	Culioli et al. 2001 [40]
methanol:chloroform (1:1)	Sterols, i.e., fucosterol	Bouzidi et al. 2008 [35]
methanol	polyphenols	Alves et al. 2016 [36]
water	polysaccharides, i.e., alginate	Gómez-Ordóñez and Rupérez, 2011 [32]
water	total dietary fibre; soluble and insoluble fibre	Gómez-Ordóñez et al. 2010 [31]
water	pholorotannins, phenmolic acids, flavonoids, fuhalols, hydroxyl-fuhalols, eckol derivatives, and rosmarinic acid	Agregán et al. 2017 [33]

**Table 3:** Assessment of lipid damage * related to quality loss in chilled hake subjected to different icing conditions **.

Chemical Parameter	Chilling Time (Days)	Icing Condition		
		CT	AQ	ET
**Free fatty acids** (mg·kg^−1^ muscle)	0	64.38 A (6.52)	64.38 A (6.58)	64.38 A (6.56)
	2	78.88 cB (1.98)	66.75 bA (1.36)	58.41 aA (2.07)
	6	94.70 cC (3.41)	87.58 bB (2.86)	70.21 aB (1.79)
	9	113.67 bD (7.19)	102.72 bC (5.44)	95.97 aC (1.89)
	13	139.50 bE (6.17)	105.88 aC (7.97)	93.23 aC (3.95)
**Peroxide value** (meq. active oxygen·kg^−1^ lipids)	0	0.14 A (0.00)	0.14 A (0.00)	0.14 A (0.00)
	2	2.28 aB (0.15)	2.69 aB (0.55)	3.75 bB (0.50)
	6	2.50 aB (0.07)	2.45 aB (0.71)	3.50 bB (0.18)
	9	3.80 aB (2.62)	2.66 aB (1.05)	7.28 bC (1.14)
	13	4.37 aB (2.67)	3.69 aB (0.95)	7.24 bC (0.57)
**Thiobarbituric acid index** (mg malondialde-hyde·kg^−1^ muscle)	0	0.13 A (0.09)	0.13 A (0.09)	0.13 A (0.09)
	2	0.35 bB (0.09)	0.17 aA (0.06)	0.26 abAB (0.06)
	6	0.36 aAB (0.15)	0.35 aB (0.10)	0.48 aD (0.05)
	9	0.37 aB (0.05)	0.51 a (0.07)	0.45 aCD (0.05)
	13	0.70 bC (0.07)	0.41 aB (0.07)	0.36 aBC (0.05)

* Average values of three replicates (*n* = 3); standard deviations are indicated in brackets. For each chilling time, different lowercase letters denote significant (*p* < 0.05) differences as a result of icing conditions. For each icing condition, different capital letters denote significant (*p* < 0.05) differences as a result of chilling time. ** Icing conditions as expressed in Table 1.

## Data Availability

Not applicable.

## Sample Availability

Samples of the compounds are not available from the authors.
